# Lymphangiogenesis: novel strategies to promote cutaneous wound healing

**DOI:** 10.1093/burnst/tkae040

**Published:** 2024-09-26

**Authors:** Yang Jian, Yanqi Li, Yanji Zhang, Mingyuan Tang, Mingfu Deng, Chenxiaoxiao Liu, Maolin Cheng, Shune Xiao, Chengliang Deng, Zairong Wei

**Affiliations:** Department of Burns and Plastic Surgery, Affiliated Hospital of Zunyi Medical University, No. 149 Dalian Road, Hui chuan District, Zunyi, Guizhou, 563003, China; Department of Burns and Plastic Surgery, Affiliated Hospital of Zunyi Medical University, No. 149 Dalian Road, Hui chuan District, Zunyi, Guizhou, 563003, China; Department of Burns and Plastic Surgery, Affiliated Hospital of Zunyi Medical University, No. 149 Dalian Road, Hui chuan District, Zunyi, Guizhou, 563003, China; Department of Burns and Plastic Surgery, Affiliated Hospital of Zunyi Medical University, No. 149 Dalian Road, Hui chuan District, Zunyi, Guizhou, 563003, China; Department of Burns and Plastic Surgery, Affiliated Hospital of Zunyi Medical University, No. 149 Dalian Road, Hui chuan District, Zunyi, Guizhou, 563003, China; Department of Burns and Plastic Surgery, Affiliated Hospital of Zunyi Medical University, No. 149 Dalian Road, Hui chuan District, Zunyi, Guizhou, 563003, China; Department of Burns and Plastic Surgery, Affiliated Hospital of Zunyi Medical University, No. 149 Dalian Road, Hui chuan District, Zunyi, Guizhou, 563003, China; Department of Burns and Plastic Surgery, Affiliated Hospital of Zunyi Medical University, No. 149 Dalian Road, Hui chuan District, Zunyi, Guizhou, 563003, China; The Collaborative Innovation Center of Tissue Damage Repair and Regeneration Medicine of Zunyi Medical University, No. 6 West Xuefu Road, Xinpu District, Zunyi, Guizhou, 563003, China; Department of Burns and Plastic Surgery, Affiliated Hospital of Zunyi Medical University, No. 149 Dalian Road, Hui chuan District, Zunyi, Guizhou, 563003, China; The Collaborative Innovation Center of Tissue Damage Repair and Regeneration Medicine of Zunyi Medical University, No. 6 West Xuefu Road, Xinpu District, Zunyi, Guizhou, 563003, China; Department of Burns and Plastic Surgery, Affiliated Hospital of Zunyi Medical University, No. 149 Dalian Road, Hui chuan District, Zunyi, Guizhou, 563003, China; The Collaborative Innovation Center of Tissue Damage Repair and Regeneration Medicine of Zunyi Medical University, No. 6 West Xuefu Road, Xinpu District, Zunyi, Guizhou, 563003, China

**Keywords:** Lymphangiogenesis, Wound healing, Growth factor, Lymphatic vessels, Skin, Inflammation, Chronic wound

## Abstract

The cutaneous lymphatic system regulates tissue inflammation, fluid balance and immunological responses. Lymphangiogenesis or lymphatic dysfunction may lead to lymphedema, immune deficiency, chronic inflammation etc. Tissue regeneration and healing depend on angiogenesis and lymphangiogenesis during wound healing. Tissue oedema and chronic inflammation can slow wound healing due to impaired lymphangiogenesis or lymphatic dysfunction. For example, impaired lymphangiogenesis or lymphatic dysfunction has been detected in nonhealing wounds such as diabetic ulcers, venous ulcers and bedsores. This review summarizes the structure and function of the cutaneous lymphatic vessel system and lymphangiogenesis in wounds. Furthermore, we review wound lymphangiogenesis processes and remodelling, especially the influence of the inflammatory phase. Finally, we outline how to control lymphangiogenesis to promote wound healing, assess the possibility of targeting lymphangiogenesis as a novel treatment strategy for chronic wounds and provide an analysis of the possible problems that need to be addressed.

Highlights

- The cutaneous lymphatic system regulates tissue inflammation, fluid balance and immunological responses. Lymphangiogenesis can promote wound healing by draining exudate and improving inflammation and immune response. Impaired lymphangiogenesis in chronic wounds like diabetic foot ulcers, venous ulcers and bedsores may be one of the reasons for delayed healing.
- Lymphangiogenesis has not received much attention in wound healing. This review summarizes the evidence of lymphangiogenesis during wound healing, discusses its mechanism and the remodeling process of the renascent lymphatic vessels, and describes methods to promote wound healing by regulating lymphangiogenesis.
- Targeted regulation of lymphangiogenesis may be a new strategy for the treatment of wound healing.

## Background

The lymphatic system is an open vessel system complementary to the vascular system [1, 2]. Although blood vessels (BVs) are crucial for the transport of oxygen, nutrients and metabolites [3], lymphatic vessels (LVs) help maintain tissue fluid balance and immunoregulation [2]. In recent years, there has been a significant improvement in our understanding of the cellular and molecular features of lymphatic development and their role in disease, leading to a shift in the traditional views of the impact of the lymphatic system on health and disease [4]. LVs have been seen as passive conduits for interstitial fluid (IF), immune cell and lipid transport [5] that are not present in the central nervous system, bones and cornea [6]. Recently however, it has been confirmed that LVs not only exist in tissues such as the central nervous system [7], heart [8, 9], bones [10] and cornea [11], but also play vital roles in the progression of various diseases, including Alzheimer’s disease, cardiovascular disease, hypertension, atherosclerosis, Crohn’s disease, glaucoma and wound healing [4, 5, 12, 13].

Multiple cells, cytokines and the extracellular matrix (ECM) participate in cutaneous wound healing [14, 15]. In recent decades, there has been great interest in the effects of angiogenesis, the inflammatory microenvironment, growth factors and infection on wound healing. Several approaches have been developed to promote wound healing by promoting angiogenesis, modulating inflammation or applying growth factors [15–22]. However, even though lymphangiogenesis was observed during wound healing as early as the 1930s [23–25], its role in wound healing remains inadequately researched [26, 27]. According to recent research, lymphangiogenesis is crucial for cutaneous wound healing [12, 28–31]. Moreover, impaired lymphangiogenesis in chronic wounds such as venous ulcers [27, 31–33], diabetic ulcers [27, 33–35] and bedsores [27, 33] contributes to delayed wound healing. Chronic wounds are characterized by chronic inflammation, tissue oedema and poor vascularization. Studies have confirmed that in the early stage of chronic wounds, insufficient production and infiltration of vascular endothelial growth factor (VEGF)-C, VEGF-A and macrophages lead to impaired lymphangiogenesis. In turn, impaired lymphangiogenesis leads to insufficient drainage of extravasated fluid and obstruction of inflammatory resolution, resulting in wound tissue oedema, increased interstitial tissue pressure and chronic inflammation, further leading to poor vascularization and delayed or absent healing [12]. Therefore, these findings suggest that lymphangiogenesis is a potential wound healing target and may provide a new approach for promoting wound healing.

This review summarizes research progress on the structure and function of cutaneous LVs. We also discuss the morphological and functional changes in LVs during wound healing and describe existing preclinical studies. Promoting lymphangiogenesis may enhance wound healing and addressing lymphangiogenesis components in wound therapy may improve wound repair.

## Review

### Structure and function of cutaneous LVs

**Figure 1:**
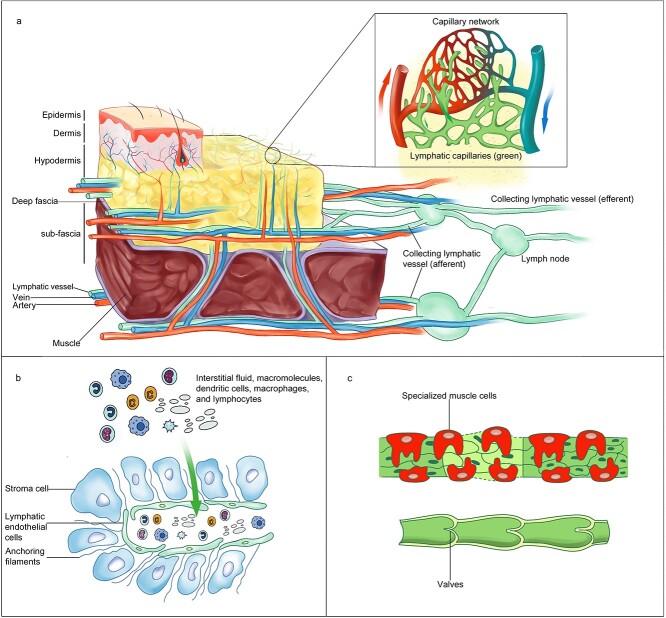
Structure of cutaneous lymphatic vessels. (**a**) Schematic diagram of the structure of the skin and lymphatic system. The lymphatic system (green) forms an arborized network parallel to the blood vasculature system (red and blue). Lymphatic capillaries absorb fluids leaked from the capillary bed and drain lymph to downstream collecting LVs and lymph nodes. (**b**) Lymphatic capillaries are composed of a single layer of loosely connected LECs and lack continuous basement membrane and perivascular mural cells, which facilitates the uptake of interstitial fluid and macromolecules, and transfer dendritic cells, macrophages and lymphocytes. (**c**) Collecting LVs are interconnected through tighter, continuous zipper-like connections between LECs and are covered with specialized muscle cells. Collecting LVs have valves that allow the unidirectional flow of lymph (below). *LVs* lymphatic vessels, *LECs* lymphatic endothelial cells

The lymphatic system is composed of lymphatic capillaries, collecting LVs, lymph nodes, lymphatic trunks and the thoracic duct (Figure 1a) [36]. The lymphatic capillaries are blind-ended, thin-walled, extremely permeable vessels that are 30–80 μm in diameter and consist of a single layer of lymphatic endothelial cells (LECs), which are not enveloped by pericytes or smooth muscle cells, lack a continuous basement membrane, and mainly absorb IF and large molecules and transport lymphocytes (Figure 1b) [4, 5, 36–38]. In contrast to the connections between vascular endothelial cells (VECs), the connections between LECs in lymphatic capillaries are noncontinuous or involve ‘button-like’ junctions [39]. Moreover, anchoring filaments, mostly emilin-1 and fibrin, connect lymphatic capillaries to the ECM (Figure 1b) [40]. Under high interstitial pressure, these filaments are operated to pull the cells and open up the overlapping junctions, widening the lymphatic capillary lumen and increasing IF absorption [36, 41].

After the IF enters the LVs, it is called ‘lymph’, which is then transported to collecting LVs with a diameter of 50–200 μm [42]. LECs in collecting LVs have tighter and more continuous ‘zipper-like’ connections, a full basement membrane and specific muscle cells that contract to assist in lymphatic flow (Figure 1c) [5, 36]. The contraction of smooth muscle cells, surrounding skeletal muscles and arterial pulsation coordinate to propel the lymph towards the proximal end [43]. Furthermore, collecting LVs have valves (Figure 1C) that prevent lymph backflow and enable unidirectional lymph flow [39, 44]. Subsequently, the lymph enters the lymph nodes via afferent LVs, where physiological processes such as antigen presentation and lymphocyte recirculation are completed, after which the lymph flow from the lymph nodes through the efferent LVs [38, 45]. Ultimately, the lymph from the left side of the body, abdomen and both lower extremities flows into the thoracic duct and flows back into the left subclavian vein connecting with the thoracic duct. The right subclavian vein, which links to the right lymphatic trunk, receives lymph from the right upper extremities, thorax and head [36, 38, 43].

LVs can regulate tissue fluid homeostasis, transport immune cells and absorb dietary fats [4, 5, 36]. Approximately 90% of the IF filtered out on the arterial side of the capillary bed is reabsorbed on the venous side. In comparison, 10% of the cells are restored to the vascular system by the lymphatic system [36]. In healthy adults, the lymphatic system returns ~1–2 l of IF daily to the venous system [36]. The absorption of dietary fats is mainly conducted by lacteal LVs in the villi of the small intestine in the form of chylomicrons [5].

In recent years, research has shown that LVs are crucial for antigen presentation, immunological and inflammatory modulation, and immune surveillance [38, 46–49]. For example, in human psoriatic plaques, LVs are dilated and tortuous [50]. Moreover, LVs expand and leak severely in skin inflammation models induced by ultraviolet B irradiation [46, 51, 52] or oxazolone [46]. On the other hand, overexpression through transgenic or viral methods or local injection of VEGF-C {the ligand of VEGF receptor (VEGFR)-3; VEGF-C/D-VEGFR-3 is the main signalling pathway regulating lymphangiogenesis [36]} can promote lymphangiogenesis and improve lymphatic drainage, thereby reducing tissue oedema and the severity of inflammation [49]. Antigen presentation, immunological activation and regulation are also mediated by LEC molecular markers [38]. For example, a chemokine expressed by LECs called CCL21 can attract CCR7^+^ dendritic cells (DCs) into LVs [38], thereby achieving antigen presentation and immune activation [53]. Moreover, the lymphatic-specific marker, lymphatic vessel endothelial hyaluronan receptor-1 (LYVE-1), can bind to clustered or polymeric hyaluronic acid on microorganisms (such as group A streptococci [54]) or cell surfaces (such as DCs [55] and macrophages [56]) to regulate their entry into LVs and migrate to draining lymph nodes to activate immune responses [57]. LVs may also be implicated in delayed wound healing [12, 27, 33], hair regeneration [13, 58, 59], skin ageing [60] and peripheral nerve regeneration [61, 62]. Although the exact mechanism is unknown, this finding implies an important role for cutaneous LVs in tissue regeneration, which may exhibit different characteristics and perform multiple specific functions depending on exposure to different environments [5, 63].

### Mechanism and remodelling of lymphangiogenesis in cutaneous wounds

Cutaneous wound healing is a complex process that involves multiple types of cells, cytokines and ECM components. These factors support haemostasis, inflammation, proliferation and remodelling [14, 15]. Here, we discuss the effect of different stages of wound healing on lymphangiogenesis.

### Hypotheses about lymphangiogenesis in cutaneous wounds

**Figure 2:**
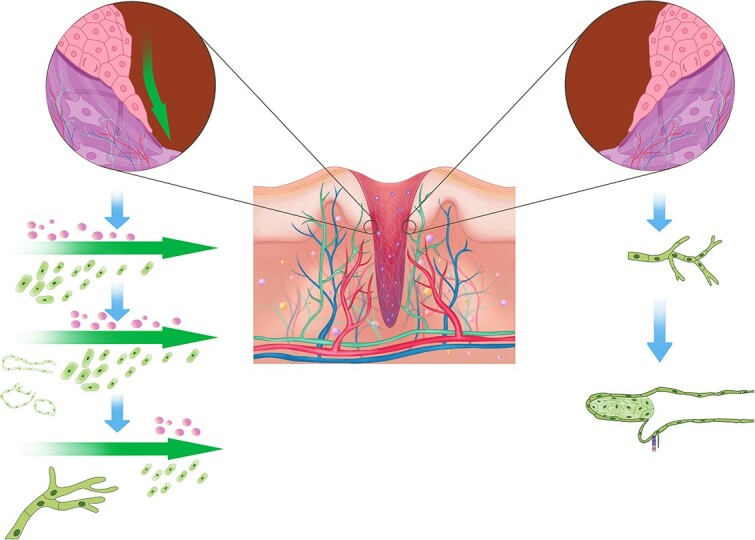
The process of lymphangiogenesis in cutaneous wound. Wound healing involves angiogenesis (red and blue), lymphangiogenesis (green), various cells and cytokines (center). Self-organization hypothesis suggests that VEGF-C is distributed along the direction of interstitial fluid flow, and LECs migrate along the direction of interstitial fluid flow (green arrow) and self-organize into lymphatic capillaries once they reach a threshold (left). Germination hypothesis suggests that residual lymphatic vessels surrounding the wound express VEGFR-3 non-uniformly and sprout gradually to form lymphatic capillaries that extend into the wound (right). *LECs* lymphatic endothelial cells, *VEGF-C* vascular endothelial growth factor-C, *VEGFR-3* vascular endothelial growth factor receptor-3

There is controversy regarding the process of lymphangiogenesis in wounds [12]. There are two main hypotheses to explain this process: the ‘self-organization’ hypothesis and the ‘germination’ hypothesis (Figure 2) [12, 64]. The ‘self-organizing’ theory states that LECs travel to the wound following IF flow and self-organize into lymphatic capillaries after reaching a numerical threshold (Figure 2, left) [12, 65]. Boardman and Swartz’s innovative skin regeneration model supported this idea by creating a circular skin wound in the centre of mouse tails, cutting off LVs that followed significant BVs and covering the wound with a collagen dermal equivalent (CDE) [66, 67]. They observed that the formation of IF drainage channels occurred before lymphangiogenesis, and the migration of LYVE-1^+^ LECs and the formation of lymphatic networks mainly occurred along the direction of IF flow [66]. Fluid channels may be formed by invading matrix metalloproteinases (MMPs) in the CDE and degrading collagen [66, 68]. Moreover, VEGF-C usually appears first at the distal (upstream) end of the CDE and is then distributed throughout the CDE, which may be why LECs migrate along the direction of IF flow [66, 68]. Conversely, reducing cutaneous IF results in the accumulation of VEGF-C and MMPs beneath the dermis of the regenerating skin and strongly inhibits lymphangiogenesis [68]. In follow-up experiments by Rutowski *et al*., they created a square excisional wound instead of a circular excisional wound in the tail of mice, which allowed lymph to bypass the regenerating area through LVs in the surrounding tissue [65]. They observed that LECs still moved with IF flow but could not organize functional LVs [65]. Moreover, the ‘self-organizing’ hypothesis is supported by *in vitro* models simulating wound-induced lymphangiogenesis [35, 64, 69]. When IF synergizes with VEGF-C, IF significantly enhances upstream lymphangiogenesis and inhibits downstream lymphangiogenesis [69].

The germination hypothesis proposes that lymphangiogenesis mainly arises from preexisting, disconnected lymphatic capillaries at the wound edge, similar to angiogenesis (Figure 2, right) [12]. In the porcine full-thickness skin wound model, sprouting of VEGFR-3^+^ vessels adjacent to BVs in granulation tissue and LVs at the wound edge was observed, implying that lymphangiogenesis during the wound healing process may occur by sprouting [27]. This phenomenon was also observed in a mouse back wound model [26, 70]. Interestingly, in an *in vitro* lymphangiogenesis model in wounds, Bianchi *et al.* [64] discovered that lymphangiogenesis in superficial wounds occurred from left to right (in the direction of interstitial flow) and could also occur from right to left. In contrast, lymphangiogenesis in deep wounds occurred only from left to right [64]. This suggests that the nature of wounds (small or deep) greatly affects lymphangiogenesis, with shallow wounds mostly undergoing growth/remodelling regardless of IF flow [12]. Indeed, in linear incisions on the backs of rats, as the wound gradually shrinks, podoplanin (PDPN)^+^ LVs in the surrounding tissue gradually develop towards the centre of the wound [71], supporting the findings of Bianchi *et al*. LVs express VEGFR-3 unevenly in wounds, suggesting that lymphatic budding may begin on the side with high expression [70]. The sprouting of LVs is also regulated by the VEGF-C/D-VEGFR-3 signalling pathway [36, 72]. In VEGF-C gene-targeted mice, recombinant VEGF-C can rescue the sprouting of LECs, with VEGF-D also having some restorative effect but to a lesser extent [72].

### Effect of different stages of wound healing on lymphangiogenesis

#### Hemostasis phase

**Figure 3:**
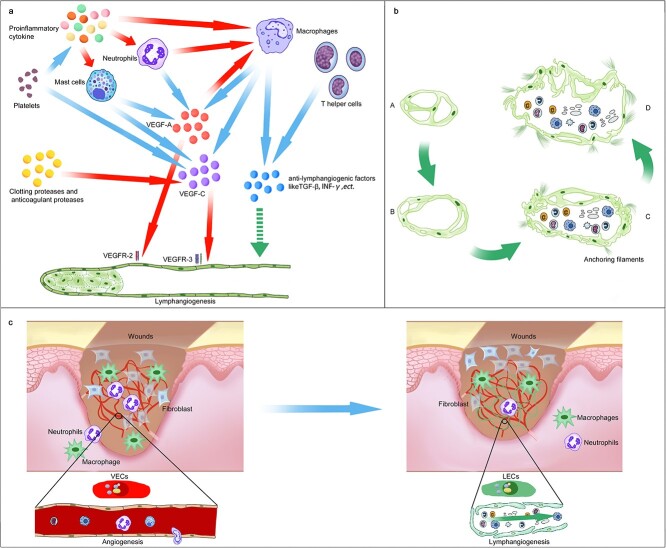
Mechanism and remodeling of lymphangiogenesis in cutaneous wound. (**a**) the mechanism of lymphangiogenesis in the wound. Red arrows indicate chemoattraction, promotion, or activation. Blue arrows indicate production. Green arrows indicate inhibition. (**b**) Schematic diagram of renascent lymphatic vessel remodeling. During the healing process, the lymphatic walls become thinner and the number of endothelial protrusions, anchoring filaments formation and interdigitating junctions increase (A,B), gradually evolving from a lymphatic-like structure to a typical lymphatic vessel (C,D), which is capable of absorbing interstitial fluid and macromolecules, as well as transporting lymph (**c**) RhoB coordinates angiogenesis and lymphangiogenesis in the wound. After skin injury, extracellular signal induces the immediate early response gene RhoB (blue circle). GTP-bound RhoB (pink circle) partially localizes in LECs and VECs and is assisted by the VEZF1 transcription complex (yellow circle) to inhibit LEC proliferation and sprouting while promoting VEC proliferation and sprouting. Angiogenesis (red) can transport nutrients, oxygen and inflammatory cells to the wound [108]. Subsequent lymphangiogenesis (green) will remove the inflammatory cells, antigens and exudate from the wound, thereby reducing swelling and inflammation. *LVs* lymphatic vessels, *LECs* lymphatic endothelial cells, *RhoB* ras homolog family member B, *GTP* guanosine triphosphate, *VEZF1* vascular endothelial zinc finger 1, *VECs* vascular endothelial cells

Haemostasis traditionally involves the formation of a fibrin clot to prepare for biological processes and provide a temporary ECM for cell migration [73]. Alternatively, recent studies have shown that haemostasis, particularly that of platelets, plays a more complex role in wound healing than previously thought [74]. Platelets are able to secrete various inflammatory mediators, including VEGF, transforming growth factor-beta (TGF-β), hepatocyte growth factor (HGF) and interleukin (IL)-8 [75, 76], thereby promoting leukocyte infiltration and angiogenesis [75, 77]. Haemostasis releases and activates VEGF-C to induce lymphangiogenesis, according to recent investigations (Figure 3a) [78]. Lim *et al*. observed that local administration of platelet-rich plasma or platelet-poor plasma immediately after severing the LVs in the mouse tail reduced the recovery of lymphatic flow by almost half [78]. *In vitro* experiments further demonstrated that clotting proteases and anticoagulant proteases activate VEGF-C and VEGF-D through protein hydrolysis and that activated platelets can stimulate the VEGFR-3 signalling pathway in LECs (Figure 3a) [78]. Furthermore, Hur *et al*. reported that PDPN^+^ monocytes (PPMs) and platelets enhance lymphangiogenesis by activating the PDPN/CLC-2 axis [79]. The culture supernatant obtained by coculturing PPMs with platelets can also enhance LEC migration and proliferation. Local injection of platelet-containing PPMs into full-thickness skin wounds in nude mice improved lymphangiogenesis and wound healing. However, some studies have shown that platelets can release anti-lymphangiogenic factors, such as TGF-β [76]. Upon the interaction of CLC-2 on platelets and PDPN on LECs, platelets can release TGF-β [80], and *in vitro* experiments have also shown that platelets can inhibit LEC migration [81]. Moreover, Sato *et al*. [82] demonstrated in a mouse model of inflammatory bowel disease that the interaction between platelets and lymphocytes exacerbates intestinal inflammation by inhibiting lymphangiogenesis. These results suggest that the role of platelets in promoting or inhibiting lymphangiogenesis depends on the stage and type of disease and the type of infiltrating cells in the surrounding tissue.

#### Inflammatory phase

Inflammation is closely related to lymphangiogenesis [49, 83]. During the acute inflammatory phase, LVs can promote exudate drainage and transport DCs and macrophages, helping to reduce tissue oedema and activate the immune response [63, 84, 85]. LV activation reduces cutaneous inflammation, while inhibition worsens it [49]. For example, VEGF-C overexpression through transgenic or viral methods [46], and local injection of VEGF-C [52, 86] can stimulate lymphangiogenesis and improve lymphatic drainage, thereby reducing tissue edema and inflammation severity. In contrast, inhibiting the interaction between VEGF-C/VEGF-D and VEGFR-3 through soluble VEGFR-3 (sVEGFR-3, an effective inhibitor of VEGF-C/VEGF-D signalling) or neutralizing antibodies can result in decreased lymphangiogenesis, tissue swelling, increased epidermal thickness, keratinocyte proliferation and increased numbers of CD8^+^ CD11b^+^ cells, leading to more severe inflammation [84, 86].

Lymphangiogenesis begins with inflammatory and growth factors [49, 63, 87, 88]. Macrophages, neutrophils and mast cells infiltrating the inflammatory microenvironment of wounds can produce various inflammatory factors [such as tumour necrosis factor (TNF)-α, TGF-β1 and IL-8] and growth factors [such as HGF, VEGF and fibroblast growth factor (FGF)] [89], which can promote or inhibit lymphangiogenesis (Figure 3a). As the first inflammatory cell population of the wound microenvironment, neutrophils [77] produce VEGF-A [90]. Hong *et al*. [91] suggested that VEGF-A above a threshold concentration may promote tissue-related lymphangiogenesis via VEGFR-2 and α1β1 and α2β1 integrins. However, some studies have shown that the overexpression or local injection of VEGF-A is insufficient to induce lymphangiogenesis [92–94]. VEGF-A is more likely to induce wound lymphangiogenesis in an environment-dependent manner [83], such as promoting lymphangiogenesis by recruiting macrophages to release VEGF-C (Figure 3a) [95, 96].

After neutrophils establish, monocytes rapidly respond to injury signals and enter the tissue, where they transform into macrophages after exposure to the local inflammatory microenvironment [77]. Macrophages are important sources of various pro-lymphangiogenic mediators, such as VEGF-C/D, TNF-α and FGF [83, 87, 97], but also secrete some anti-lymphangiogenic factors, such as TGF-β and interferon (INF)-γ (Figure 3a) [97–99]. In a skin inflammation model, K14-VEGF-C mice showed expansion of dermal LVs, reduced tissue swelling and less redness. However, the depletion of macrophages induced by clodronate led to decreased lymphangiogenesis and delayed the resolution of inflammation [84]. Moreover, high glucose-treated macrophages downregulated VEGFR-3 and VEGF-C/D. However, macrophages derived from db/db diabetic mice treated with IL-1β induced lymphangiogenesis in full-thickness skin wounds of db/db mice, accelerating wound healing [34]. Similar results were obtained in a myocardial infarction model [100]. Cd36^−/−^ mice exhibited decreased VEGF-C expression in cardiac macrophages and decreased LYVE-1 and VEGF-C expression in the myocardium following myocardial infarction. Myeloid-derived VEGF-C deficiency also increased the acute infarction area [100]. Conversely, *in vitro* experiments have shown that adding apoptotic cardiomyocytes to primary cardiac macrophages promotes VEGF-C expression [100]. Moreover, *in vitro* experiments by Maruyama *et al*. [101] demonstrated that CD11b^+^ macrophages alone can form tubular structures and express lymphatic markers such as LYVE-1 and PDPN. Interestingly, in excisional wounds, lymphangiogenesis in the early stages involves cells co-stained with the macrophage marker F4/80 and the lymphatic markers LYVE-1 and PDPN [34]. These results suggest that macrophages promote lymphangiogenesis by secreting lymphangiogenic factors. Nevertheless, they may also be a source of lymphatic endothelial progenitor cells by differentiating into LECs and being incorporated into growing LVs [102].

Macrophages can also secrete some anti-lymphangiogenic factors, such as TGF-β and INF-γ (Figure 3a) [97–99]. In a mouse full-thickness tail skin wound model, TGF-β1 hindered LEC proliferation and function and caused lymphatic fibrosis, while covering the wound with a collagen gel significantly reduced TGF-β1 and decreased scar/fibrosis, accelerating lymphangiogenesis [103]. Similar results were obtained by Avraham *et al*. using a mouse tail lymphedema model [104]. Blocking TGF-β with a monoclonal antibody or by topically administering soluble faulty TGF-β receptors enhanced wound healing, oedema and tissue fibrosis compared to those in the control group [104].

Mast cells and T lymphocytes in the inflammatory phase of wounds also impact lymphangiogenesis. Mast cells can promote lymphangiogenesis directly or indirectly by secreting histamine, VEGF-C/D and TNF-α (Figure 3a) [77, 88]. Different subsets of T lymphocytes exhibit differences in cytokine release in skin wounds [77]. For example, cytokines such as INF-γ from T helper (Th) 1 cells and IL-4, IL-13 and TGF-β from Th2 cells have all been shown to have strong anti-lymphangiogenic activity and impair LEC proliferation, differentiation and migration (Figure 3a) [98]. Regulatory T cells (Tregs) have been shown to regulate inflammation in wounds, and limited or depleted accumulation of Tregs impairs the early inflammatory stage of wound healing [77]. Unfortunately, the direct effects of these T-cell types on wound healing are still unknown and their different functions need to be further clarified.

#### Proliferative phase

The proliferation phase is characterized by granulation tissue formation, angiogenesis, lymphangiogenesis, collagen deposition and epithelialization [12, 15]. Angiogenesis supplies nutrients and oxygen for granulation tissue and epithelialization [21]. Indeed, lymphangiogenesis occurs slightly later than angiogenesis [27, 34, 65, 105]. Using VEGFR-3 as a marker for LECs, lymphangiogenesis occurs 4–5 days after damage [27, 34], somewhat later than angiogenesis [27, 34, 105]. The LVs density peaks at ~7 days [63, 70, 106]; at this point, LVs and lymphatic flow can be observed through near-infrared fluorescence imaging [106]. Increased angiogenesis and vascular permeability provide oxygen and nutrients for LECs [12, 70] and create an IF microenvironment [107]. Lymphangiogenesis may occur later than angiogenesis during wound healing because of the regulation of the transcription factor vascular endothelial zinc finger 1 (VEZF1) by nuclear ras homologue family member B-guanosine triphosphate (RhoB-GTP), which controls the expression of different gene sets in each endothelial lineage (Figure 3c) [108]. RhoB-GTP and VEZF1 stimulate VEC proliferation and sprouting but inhibit LEC proliferation and sprouting, coordinating early and delayed lymphangiogenesis. Delayed lymphangiogenesis allows inflammatory cells time to initiate tissue repair [12, 108].

The proliferation phase also involves the proliferation and differentiation of numerous stem cells, including hair follicle stem cells (HFSCs), which are involved in hair and epidermal regeneration [73]. Recently, Yoon *et al*. [59] discovered through fluorescence stereomicroscopy that the lymphatic network of mice directly connects to individual hair follicles (HFs) and that CD31^+^/LYVE-1^+^ LVs are near HFs in the early anagen phase, mainly near the bulge region of HFs. In the hair regeneration model in K14-VEGF-C mice, the mRNA expression levels of VEGF-C and VEGFR-3 in the dorsal skin were significantly greater during the anagen phase than during the catagen phase, and the LV density in the skin increased during the early anagen phase, which accelerated the growth of HFs. HF degeneration is accelerated in K14-sVEGFR3 transgenic mice lacking cutaneous LVs [59]. By using static and live imaging approaches, Peña-Jimene *et al*. [58] also reported that LVs were primarily near the bulge region of HFs and connected adjacent HFs in a stem cell-dependent manner. LVs briefly dilate, enhancing tissue drainage capacity when HFSCs are activated or HFs development is induced, but lymphatic depletion blocks HF growth [58]. However, Gur-Cohen *et al*. reported that drainage was reduced, although growing HFs were associated with highly expanded lymphatic capillaries [13]. In the absence of lymphatic capillaries and restricted lymphatic drainage, quiescent HFSCs are activated and HFs enter the anagen phase, while the lymphatic capillaries around HFs remodel and shift away from the stem-cell-rich bulge area. The authors further demonstrated that resting HFSCs express high levels of angiopoietin-like (Angptl)-7, while growing HFSCs express Angptl-4 and Netrin-4 [13]. When HFs are damaged, the expression of Angptl-7 rapidly decreases, activating HFSCs to repair the damage. After activation, HFSCs switch to Angptl-4 and Netrin-4, leading to transient lymphatic dissociation and reduced drainage, which triggers HFs to enter the anagen phase [13]. In conclusion, HF regeneration and LVs are linked but this remains a contentious issue. Clarification on how LVs affect HF regeneration and wound healing is needed.

#### Remodeling phase

Renascent LVs undergo remodelling similar to that of renascent BVs once they have grown to a certain extent [70]. However, unlike vascular remodelling, the remodelling of renascent LVs in wounds has not been well described. In the mouse tail skin regeneration model, lymphangiogenesis usually occurs on day 10, and remodelling is completed within 60 days [65, 66], later than in full-thickness dorsal skin wound models. Initially, the recurrent LVs are lymphatic-like structures with incomplete lumens (Figure 3b) [70]. LECs have simple intercellular connections that allow IFs, inflammatory cells and other macromolecules to enter LVs as the lumen expands (Figure 3b) [27, 63, 70]. Subsequently, the lymphatic wall becomes thinner and more irregular and LECs protrude into the lymphatic lumen (Figure 3b). Intercellular connections overlap and become interlaced, and protrusions into the lymphatic lumen and matrix increase (Figure 3b). The renascent LVs gradually extended into the superficial dermis [70]. The mechanisms of lymphatic remodelling in wounds are still not fully understood [109], but they may be related to collagen and fibronectin [109], MMPs [66, 68], integrins [5, 91, 109] and hyaluronic acid [109]. Interestingly, fibrosis decreases during wound healing as lymphangiogenesis and lymphatic drainage increase [110], but lymphatic capillary walls thicken in fibrotic wounds [109]. Ji *et al*. [70] reported that fibroblasts and mesenchymal progenitor cells appear around neonatal LVs during lymphatic remodelling. Although the specific role of these cells is unknown, they may be necessary for lymphatic remodelling.

In conclusion, lymphangiogenesis is closely linked to wound healing, specifically the inflammatory stage, during which the interaction between inflammatory cells and LVs affects the healing process [49, 83]. Clarifying lymphangiogenesis and renascent LV structural and functional changes during chronic wound healing may assist in the development of innovative wound healing therapies [12].

### Lymphangiogenesis strategies for promoting cutaneous wound healing

As the role of lymphangiogenesis in wound healing has become increasingly evident in recent years, targeting lymphangiogenesis may be a new field for promoting wound healing [12]. Here, we summarize therapeutic approaches for regulating lymphangiogenesis to promote wound healing (Table 1).

**Table 1:** Existing strategies for regulating lymphangiogenesis to promote wound healing in animal models

Study	Animal model	Intervention	Control	Outcome
**Growth factors**				
Saaristo *et al*. 2006 [26]	Full-thickness skin wound model on the back of db/db diabetic mice	Local injection of adenovirus encoding VEGF-C, VEGF-C156S, VEGF-A165, soluble VEGFR-2-Ig and soluble VEGFR-3-Ig	Local injection of adenovirus encoding VEGF-B186 or LacZ	1. VEGF-C, VEGF-C156S and VEGF-A all accelerate wound healing, but wounds treated with VEGF-A healed faster at 7 and 17 days compared to wounds treated with VEGF-C or VEGF-C 156S. 2. Both sVEGFR-2-Ig and sVEGFR-3-Ig significantly delay wound healing, while respectively inhibiting angiogenesis and lymphangiogenesis. 3. VEGF-C enhances angiogenesis and lymphangiogenesis in the wounds. 4. VEGF-C promotes the recruitment of hematopoietic cells and macrophages to the wound edge
Saaristo *et al*. 2004 [111]	NMRI nu/nu mouse with a pedicle flap in the abdominal region	Local injection of adenovirus encoding VEGF-C and VEGF-C156S	Local injection of adenovirus encoding LacZ	1. VEGF-C or VEGF-C156s can promote lymphangiogenesis in flaps and their connection with the surrounding lymphatic network. 2. VEGF-C can promote functional recovery of lymphatic vessels.
Güç *et al*. 2017 [115]	Mouse ear subcutaneous cartilage-replacement healing model; full-thickness skin defect model on the backs of diabetic mice	FB-VEGF-C hydrogel covering the wound	Hydrogel containing free VEGF-C covering the wound	1. FB-VEGF-C is stable and slowly releases VEGF-C while inducing lymphatic capillary and remodeling with interstitial flow synergistically, but does not induce downstream collecting lymphatic vessel remodeling. 2. Dendritic cells migration is enhanced in the lymphangiogenic regions, but fluid clearance rate is not affected. 3. FB-VEGF-C promotes diabetic wound healing while simultaneously enhancing CCL21 and leukocyte trafficking in diabetic wounds.
Brunner *et al*. 2023 [31]	Full-thickness skin wound on the backs of diabetic mice	Intravenous injections of F8-VEGF-C into the tail vein	Intravenous injections of F8-SIP into the tail vein	1. Increased lymphangiogenesis. 2. Inflammatory cell infiltration decreased. 3. Increased fibroblast density and type I collagen deposition.
Hall *et al*. 2013 [106]	Full-thickness skin incision between the inguinal and axillary	Local injection of VEGF-C	0.85% NaCl	1. VEGF-C promotes lymphangiogenesis, increasing the number of lymphatic vessels, the number of branches, their length and the connectivity between branches. 2. Wounds treated with VEGF-C showed a more significant average response in cytokine/chemokine expression. IL-1α and IL-22 levels were significantly increased on day 7 post-injury compared to their respective pre-injury levels
Hong *et al*. 2004 [91]	Full-thickness skin wound on the backs of mice	Overexpression of VEGF-A	Wild-type littermates of transgenic mice	1. Prolonged up-regulation of VEGF-A and VEGFR-2 mRNA expression during wound healing in VEGF-A transgenic mice. 2. Increased angiogenesis and lymphangiogenesis in the granulation tissue of VEGF-A transgenic mice.
Cho *et al*. 2006 [117]	Full-thickness skin wound on the tails of db/db diabetic mice	Tail vein injection of adenovirus encoding COMP-Ang-1 or local injection of recombinant COMP-Ang-1 protein	Tail vein injection of adenovirus encoding LacZ or local injection of bovine serum albumin	1. COMP-Ang1 promotes angiogenesis, lymphangiogenesis, blood flow and wound healing. 2. Topical COMP-Ang1 promotes wound healing with enhanced angiogenesis and blood flow in tail skin.
**RNA**				
He *et al*. [118]	Full-thickness skin wound on the backs of diabetic mice	Local injection of lncRNA-GAS5 adenovirus particles	Local injection of vehicle PBS	1. lncRNA-GAS5 up-regulated the expressions of VEGFR-3, LYVE-1 and Prox1. 2. lncRNA-GAS5 could save the damage and apoptosis of LECs cell viability, migration and tube-forming ability caused by high glucose. 3.lncRNA-GAS5 overexpression accelerated wound healing and promoted lymphangiogenesis in diabetic mice
He *et al*. [119]	Full-thickness skin wound on the backs of diabetic mice	Local injection of ANRIL adenovirus particles	Local injection of vehicle PBS	1. ANRIL up-regulated Prox1 expression. 2. ANRIL could save the damage and apoptosis of LECs viability, migration and tube-forming ability caused by high glucose. 3. Overexpression of ANRIL rescued the impairments of survival, migration, epithelial–mesenchymal transition formation and tube formation of LECs caused by high glucose.
**Simvastatin and herbal**				
Asai *et al*. 2012 [122]	Full-thickness skin wound model on the backs of db/db diabetic mice	Topical application of simvastatin	Petroleum jelly	1. Simvastatin accelerates diabetic wound healing via promoting both angiogenesis and lymphangiogenesis. 2. Simvastatin induces capillary morphogenesis of LECs and has an antiapoptotic effect but does not induce proliferation. 3. Simvastatin promotes macrophage infiltration and VEGF-C production in wounds.
Avraham *et al*. 2010 [104]	Mouse tail lymphedema model with circumferential full thickness skin excision 2 mm wide	Intraperitoneal injection of anti-mouse TGF-β mab or wounds covered with 1% collagen gel containing adenovirus encoding soluble dominant negative TGF-β type II receptor	Intraperitoneal injection of antibody or wounds covered with 1% collagen gel containing adenovirus encoding LacZ or recombinant human TGF-β1	1. Treatment with TGF-β mab improved animal wound healing and resulted in mild edema. 2. TGF-β1 blockade improves lymphatic function, decreases tissue fibrosis and reduces lymphedema-induced chronic inflammation.
Choi [123]	Full-thickness skin wound on the backs of mice	Topical application of herbal mixture composed of *Alchemilla vulgaris* and *Mimosa*	Topical application of fusidic acid	1. Significant reduction of wound size treated with herbal mixture. 2. Increased F4/80 macrophage infiltration and number of LYVE-1^+^ lymphatic vessels.
**Stem cells**				
Zhou et al. 2021 [28]	Full-thickness skin wound on the backs of diabetic mice	Diabetic ulcer mice were locally injected with ADSCs, ADSCs-sh-METTL3 or ADSCs-sh-IGF2BP2	Normal mice with no treatment, diabetic ulcer mice with no treatment and diabetic ulcer mice locally injected with ADSCs-sh-NC.	1. ADSCs accelerate LECs proliferation, migration and lymphangiogenesis via METTL3 pathway. 2. ADSCs regulate VEGF-C expression through the METTL3/IGF2BP2 m^6^A pathway, enhancing VEGFR3-mediated lymphangiogenesis and thus promoting diabetic ulcer wound healing. 3. Both METTL3 knocked-down and IGF2BP2 knocked-down ADSCs suppressed the wound healing rate in diabetic ulcer mice.
Yamada *et al*. 2022 [131]	Full-thickness skin wound on the backs of diabetic mice	Implanted with 1 × 10^6^ npBM-MSCs	Implanted with 1 × 10^6^ mBM-MSCs	1. npBM-MSC transplantation promoted wound healing in diabetic mice. 2. Xenotransplantation of npBM-MSCs promoted both angiogenesis and lymphangiogenesis in wound healing. 3. Xenotransplantation of npBM-MSCs enabled and contributed to angiogenesis and lymphangiogenesis in the early posttransplant period.
Beerens *et al*. 2018 [132]	Full-thickness skin wound on the backs of mice; a pedicled abdominal flap model of athymic nude Foxn1 mice	Local injection of MAPCs	Local injection of PBS	1. MAPCs possess the potential for lymphangiogenesis and angiogenesis, and can promote lymphatic capillary growth in wounds, thus promoting wound healing. 2. MAPCs accelerate lymphatic capillary and collecting lymphatic vessels restoration in skin flaps.
Lee *et al*. [133]	Full-thickness excisional skin wounds on backs of male athymic nude mice and ear wound model of mice	Local injection of hPSC-derived LYVE-1^+^ Podoplanin^+^ cells (1 × 10^5^)	Local injection of PBS or hLECs	1. hPSC-derived LYVE-1^+^ Podoplanin^+^ cells contribute to lymphatic vessel formation. 2. hPSC-derived LYVE-1^+^ Podoplanin^+^ cells promote wound healing through lymphatic neovascularization.
**Biological materials**				
Marino *et al*. 2014 [136]	Full-thickness skin wound on immunoincompetent nu/nu rats	Collagen hydrogel with HDMECs (bioengineered human dermo-epidermal skin grafts)	Collagen hydrogel combined with fibroblasts	1. Tissue-engineered skin can form blood vessels and functional lymphatic capillaries and remain stable and persistent. 2. Compared to the control group, tissue-engineered skin’s lymphatic capillaries can anastomose with host animal lymphatic vessels and drain fluid.
Kong *et al*. [137]	Full-thickness skin wound on the backs of male nude rats	3D Porous scaffold with hLECs + hvSMC	3D Porous scaffold with hLECs or hBECs ± hvSMC	hLECs group also demonstrated significantly increased individual vessel and network size, and longer survival than hBECs capillaries *in vivo*, and established inosculation with rat lymphatics, with evidence of lymphatic function.
Jiang *et al*. [138]	Full-thickness skin wound on the backs of immunocompromised mice	Topical application of 40% HPS	Topical application of 10% HPS	1. HPS accelerates wound closure. 2. HPS-40% promotes angiogenesis and lymphangiogenesis.
Frueh *et al*. 2017 [139]	Full-thickness skin wound on the backs of mice	Collagen-glycosaminoglycan matrix with adipose-derived ad-MVF covering the wound	Collagen-glycosaminoglycan matrix covering the wound	1. Treatment with ad-MVF significantly increased collagen content in the wound, accelerated epithelialization and improved wound healing. 2. Treatment with ad-MVF increased functional lymphatic vessel density and blood flow perfusion in the wound bed, and the microvascular network of the graft was interconnected with that of the surrounding host microvasculature system.
Frueh *et al*. [140]	Full-thickness skin defect on the crowns of the skulls of CD1 nu/nu mice	1. Integra matrices loaded with ad-MVF 2. Integra matrices loaded with ad-MVF + STSG	1. Integra matrices 2. Integra matrices + STSG	1. STSG on prevascularized Integra exhibited a survival rate of 40% ± 11%, significantly higher than that of the control group. 2. Integration of the prevascularized matrices was markedly enhanced. 3. Lymphatic vessel and microvessel density of prevascularized implants was markedly increased.

*ad-MVF* adipose tissue-derived microvascular fragments, *ADSCs* adipose derived stem cells, *ANRIL* long noncoding RNA-antisense noncoding RNA in the inhibitor of cyclindependent kinase 4 locus, *CCL21* chemokine (C-C motif) ligand 21, *CD1* cluster of differentiation 1, *COMP-Ang-1* cartilage oligomeric matrix protein angiopoietin-1, *FB-VEGF-C* fibrin-binding variant of vascular endothelial growth factor C, *F8-VEGF-C* a fusion protein consisting of human VEGF-C fused to the F8 antibody, *F8-SIP* F8-small immunoprotein; *F4/80* mouse epidermal growth factor-like module-containing mucin-like hormone receptor-like 1, *hBECs* human blood endothelial cells, *HDMECs* human dermal microvascular endothelial cells, *hLECs* human lymphatic endothelial cells, *HPS* hypoxia preconditioned serum, *hPSC* human pluripotent stem cells, *hvSMC* human vascular smooth muscle cells, *IL* interleukin, *LacZ* β-galactosidase, *LECs* lymphatic endothelial cells, *lncRNA-GAS5*, long noncoding RNA GAS5, *LYVE-1* lymphatic vessel endothelial hyaluronan receptor 1, *MAPCs* multipotent adult progenitor cells, *mBM-MSCs* mouse bone marrow mesenchymal stem cells, *METTL3* methyltransferase-like 3, *IGF2BP2* insulin-like growth factor 2 binding protein 2, *npBM-MSCs* neonatal porcine bone marrow mesenchymal stem cells,* NMRI* Naval Medical Research Institute, *PBS* phosphate buffered saline, *Prox1* ﻿prospero homeobox 1, *RNA* ribonucleic acid, *STSG* split-thickness skin grafts, *sVEGFR-2-Ig* soluble VEGFR-2-Ig, *TGF* transforming growth factor, *TGF-β mab* TGF-β monoclonal antibody, *VEGF* vascular endothelial growth factor

#### Drugs

VEGF-C, a classical growth factor that induces lymphangiogenesis [36], was initially considered to promote wound healing. In full-thickness skin wounds in diabetic mice, VEGF-C overexpression by adenovirus promoted angiogenesis and lymphangiogenesis, accelerating wound healing [26]. In contrast, wound closure was significantly delayed after VEGF-C was blocked [26]. Earlier studies by Saaristo *et al*. also showed that in an Naval Medical Research Institute (NMRI) nu/nu mouse abdominal flap model, injecting adenoviruses encoding VEGF-C and VEGF-C156S into the entire flap edge promoted anastomosis between the flap LVs and surrounding LVs, and continuous lymphatic function was detected even 2 months after surgery [111]. In the control group, only a few nonfunctional renascent LVs were found 2 months after surgery [111]. However, when using viral vectors to deliver VEGF-C-mRNA, it is difficult to control the dosage, and this approach carries the risk of unpredictable immune responses [112]. Moreover, direct application of VEGF-C results in a short duration of action, and high doses released in a short period may result in pathological vascular proliferation, increased permeability and lymphatic dysfunction [113,114]. Güç *et al*. [115] recently introduced engineered fibrin-binding VEGF-C to overcome these issues. Notably, local lymphangiogenesis is stimulated in a dose-dependent manner by fibrin-binding VEGF-C, which can improve granulation tissue formation and promote diabetic mouse wound healing by enhancing leukocyte transport without affecting downstream LVs collection [115]. Moreover, Brunner *et al*. [31] used the F8-VEGF-C (a fusion protein consisting of human VEGF-C fused to the F8 antibody) fusion protein to specifically deliver VEGF-C to inflammatory sites, promoting wound healing by increasing lymphatic density, myofibroblast density and type I collagen deposition and reducing inflammatory cell infiltration in diabetic wounds. However, excess VEGF-C promoted LEC proliferation and lymphangiogenesis but did not affect lymphatic diameter or density in a mouse tail skin regeneration model [116]. Furthermore, Hall *et al*. reported that although injecting VEGF-C around linear incisions in mice increased the number of lymphatic branches and connections, these changes were not significant during the wound healing process [106]. Thus, the VEGF-C expression pattern and timing must be clarified in specific types of wounds to guide its use.

VEGF-A induces the production of α1β1 and α2β1 integrins through VEGFR-2, facilitating lymphatic vessel regeneration in wounds [91]. In full-thickness skin wounds on the backs of transgenic mice overexpressing VEGF-A, there was an increase in wound angiogenesis, with LYVE-1^+^ LVs detected on day 7 and an increase in the number of enlarged LVs observed in the first 3 weeks after injury. In contrast, no LVs were observed in wild-type mice on day 7 and only a few sprouting LVs were observed by day 10 [91]. However, wild-type mice showed no acceleration of wound closure and only a minor delay in the later stages of wound healing, presumably due to their decreased wound size [91]. In contrast, Saaristo *et al*. [26] reported that injecting an adenovirus encoding VEGF-A around full-thickness skin wounds in diabetic mice promoted wound healing. Compared with those treated with VEGF-C or VEGF-C156s, wounds treated with VEGF-A healed earlier, at 7 and 17 days after injury. However, some studies have also shown that VEGF-A overexpression or local injection cannot induce lymphangiogenesis [92–94], and it is more likely that VEGF-A promotes lymphangiogenesis by inducing the recruitment of inflammatory cells that release VEGF-C [95, 96].

Angiopoietin-1 (Ang-1), a unique growth factor that promotes stable and functional vascularization through the tyrosine kinase receptors with immunoglobulin and epidermal growth factor homology domain receptors 2 (Tie 2) and Tie1, promotes wound healing in diabetic mice [12]. Cho *et al*. studied cartilage oligomeric matrix protein (COMP)-Ang1, a soluble, stable and effective form of Ang1, and evaluated its efficacy in promoting wound healing in diabetic mice [117]. They found that mice treated with COMP-Ang-1 exhibited increased angiogenesis and lymphangiogenesis, accelerated wound healing and faster regeneration of the epidermis and dermis [117].

These findings indicate that growth factors play a multifaceted role in wound healing and vary in efficacy. Appropriate growth factors must be secreted at the correct time and at a precise concentration to achieve good results. Our inadequate understanding of how growth factors interact during wound healing and the low efficacy of single growth factor therapies in chronic wounds limit their practical application [20].

Promoting lymphangiogenesis through RNA delivery is also a novel strategy to promote chronic wound healing. He *et al*. [118] reported that overexpression of the long noncoding RNA GAS5 (lncRNA-GAS5) could reduce high-glucose-induced damage to and apoptosis of LECs, as well as changes in cell viability, migration and tube-forming ability, and the expression of VEGFR-3, LYVE-1 and ﻿prospero homeobox 1 was increased. In the wounds of diabetic mice, lncRNA-GAS5 can upregulate ﻿prospero homeobox 1 and promote lymphangiogenesis and wound healing. Those authors also studied the effect of lncRNA-antisense noncoding RNA in the inhibitor of cyclindependent kinase 4 locus (ANRIL) on the wounds of diabetic mice and obtained results similar to those reported for lncRNA-GAS5 [119]. Although studies have shown that the delivery of RNA may promote lymphangiogenesis and wound healing, its effects may be multifaceted. Moreover, uncertainties related to the rate and amount of degradation of the delivered RNA limit its application. Therefore, more studies are needed to explore the effects of these RNAs on lymphangiogenesis in chronic wounds.

In addition to growth factors, some drugs have also been shown to promote lymphangiogenesis in wounds [12]. Simvastatin, a 3-hydroxy-3-methylglutaryl coenzyme A reductase inhibitor, has been shown to promote wound healing in diabetic wounds through its ability to stimulate lymphangiogenesis in animal experiments [120,121]. In diabetic mice, local simvastatin administration increases angiogenesis and lymphangiogenesis, promoting wound healing [122]. Moreover, *in vitro* experiments have shown that simvastatin can promote LEC tube formation and has an antiapoptotic effect on LECs. It can also promote macrophage infiltration and stimulate macrophages to secrete VEGF-C [122]. These findings suggest that simvastatin may be an effective drug for promoting lymphangiogenesis and wound healing. Furthermore, lymphangiogenesis can also be promoted by blocking anti-lymphangiogenic factors. Experiments have shown that blocking the TGF-β signalling pathway through anti-TGF-β monoclonal antibodies can promote lymphangiogenesis during wound healing, reduce oedema and improve wound repair [104]. Some herbal extracts have been shown to increase lymphangiogenesis in wounds; e.g. an herbal mixture composed of *Alchemilla vulgaris* and *Mimosa tenuiflora* has been shown to enhance the migration of keratinocytes, fibroblasts and endothelial cells and promote the proliferation of macrophages and lymphocytes in the back wounds of mice [123]. Unfortunately, the available research has primarily involved animal experiments and evidence from clinical trials is lacking. However, the benefits of these drugs for wound healing need further study.

#### Stem cells

The migration, proliferation and differentiation of stem cells, including mesenchymal stem cells (MSCs) from HFs, damaged nerves and bone marrow, are important components of wound healing [89]. Several clinical and preclinical studies have evaluated the therapeutic potential of using stem cells in lymphangiogenesis [124]. The mechanism by which stem cells promote lymphangiogenesis may include [125–127]: (1) implanted stem cells release pro-lymphangiogenic factors, including VEGF-C, that might stimulate the migration and proliferation of residual LECs and eventual lymphangiogenesis; (2) released cytokines could enhance the mobilization and/or recruitment of bone marrow-derived M2 macrophages as lymphatic endothelial progenitors; and (3) stem cells directly transdifferentiate into mature LECs.

Indeed, studies have shown that MSCs-conditioned medium or co-culture with MSCs can significantly promote LEC proliferation, migration and tube formation [128]. Intriguingly, LECs supernatant can induce the expression of lymphatic markers and transform MSCs into an endothelial-like morphology *in vitro* in human and mouse MSCs [129]. Moreover, MSCs showed migratory activity along the VEGF-C gradient, which enhanced the migratory activity regulated by VEGF-C [129]. MSCs reduced oedema and restored methylene blue drainage in a mouse tail lymphedema model after 7 weeks [129]. Further research has shown that adipose-derived mesenchymal stem cells promote the proliferation, migration and tube formation of LECs via the methyltransferase like 3 pathway and regulate VEGF-C expression through the methyltransferase like 3/insulin-like growth factor 2 binding protein 2-m^6^A pathway, thus enhancing VEGFR-3-mediated lymphangiogenesis and promoting full-thickness skin wound healing in diabetic mice [28]. *In vitro*, adipose-derived mesenchymal stem cells were cocultured with LECs under hypoxic conditions, VEGF-A, VEGF-C, VEGFR-2 and VEGFR-3 were significantly overexpressed, and their protein expression profiles and angiogenic and lymphangiogenic potential were enhanced [130]. Neonatal porcine bone marrow MSCs (npBMMSCs) and mouse BMMSCs (mBMMSCs) were transplanted into the full-thickness skin wounds of diabetic mice, and the rates of wound healing, angiogenesis and lymphangiogenesis in the npBMMSC group were significantly greater than those in the control group and mBMMSC group. The secretion of VEGF-A, VEGF-C and TGF-β by npBMMSCs was significantly greater than that by mBMMSCs [131]. This is consistent with the treatment of lymphedema by stem cells, which can promote lymphangiogenesis via paracrine lymphocytic regenerative factors such as VEGF-C and VEGF-A. Furthermore, multipotent adult progenitor cells can stimulate LEC sprouting, proliferation and migration *in vitro* [132]. Local application of multipotent adult progenitor cells significantly increases the growth of LYVE-1^+^ or PDPN^+^ lymphatic capillaries in full-thickness skin wounds in mice, accelerates wound healing, leads to smaller scars and promotes the reconnection of LVs in the skin flap and surrounding tissues, restoring lymphatic drainage and reducing postoperative swelling after skin flap surgery [132]. Lee *et al*. [133] demonstrated that LECs can be selectively isolated from differentiated human pluripotent stem cells and from full-thickness excisional skin wounds in nude mice. Compared with control or human LEC (hLEC)-derived LECs, human pluripotent stem cell-derived LECs improved wound healing and increased lymphangiogenesis. This means that stem cells may differentiate into LECs and promote lymphangiogenesis in wounds, but the mechanisms by which stem cells promote lymphangiogenesis during wound healing have not been well studied.

#### Biological materials

The design of appropriate materials based on the pathophysiological conditions of wounds to promote wound healing has received significant attention [14, 15]. In recent years, several tissue engineering strategies aimed at promoting lymphangiogenesis have been developed [134]. Functional lymphatic drainage contributes to the successful engraftment of dermal substitutes, and therapeutic lymphangiogenesis is an emerging field of research in skin reconstruction [135]. Marino *et al*. [136] created a hydrogel dermal-epidermal skin graft that regenerates BVs and LVs utilizing fibronectin hydrogel and human dermal microvascular endothelial cells, a mixture of endothelial cells from dermal BVs and LVs. The graft was transplanted onto the backs of immune-deficient nu/nu rats for 14 days, and confocal microscopy revealed a match between the transplanted graft and host animal lymphatic capillaries, with the characteristic features of ‘direct connection’ or ‘encircling connection’ between LVs. Moreover, compared to the control group (transplanted with fibronectin hydrogel combined with fibroblasts), the experimental group showed increased absorption and drainage of Evans blue dye 30 min after injection, indicating the existence of functional lymphatic connections between the graft and the host animal [136]. Kong *et al*. [137] compared the effects of 3D porous scaffolds implanted in hLECs and hVECs on lymphangiogenesis and angiogenesis *in vivo* and *in vitro*, respectively. They found that the capillary percent vascular volume and vascular density were significantly greater in the hLECs group. The hLEC group also exhibited significantly greater individual vessel and network sizes and longer survival than the human blood endothelial cells group *in vivo*, and the hLEC group was established via inosculation with rat lymphatic vessels, with evidence of lymphatic function. Another strategy for promoting lymphatic regeneration is hypoxia preconditioning of hydrogels. Jiang *et al*. [138] prepared alginate gels loaded with 10 and 40% hypoxia-preconditioned serum (HPS) and used them to treat wounds in mice. Compared with those in the control group, both of these treatments promoted wound healing, but in the HPS-40% group, the number of blood vessels in the LYVE-1^+^ LVs group increased by 45% at 15 days after treatment, and the number of blood vessels in the HPS-40% group was similar to that in the control group.

In addition to human dermal microvascular endothelial cells, adipose tissue-derived microvascular fragments isolated from adipose tissue may also be suitable for generating lymphatic networks in skin substitutes [139]. Compared to nonimplanted grafts, transplanting tissue-derived microvascular fragments-containing grafts into full-thickness skin wounds in mice for 14 days increased the lymphatic and vascular density and created microvascular networks with the surrounding tissue, accelerating epithelialization and wound healing [139]. Similar results were obtained by Frueh *et al*. [140], who also reported that skin graft necrosis occurred in the control group on day 10 after surgery. However, like most skin substitutes, this lymphatic and vascularized skin substitute requires further research to evaluate its effectiveness.

Dressings designed to promote lymphangiogenesis have also been developed and validated *in vitro*. For example, Chavez *et al*. [141] developed a photosynthetic scaffold loaded with a transgenic *Synechococcus sp. PCC 7002* cyanobacterium that produces oxygen and hyaluronic acid, which has been shown to promote LEC proliferation and lymphangiogenesis under hypoxic conditions. However, these methods have only been successful in *in vitro* experiments and their effectiveness needs to be further evaluated.

**Other methods**

Moreover, wounds treated with negative pressure wound therapy [29, 30, 33, 142] and acellular dermal matrix [143, 144] have shown an increase in LV density and diameter, which may aid in wound healing. In addition, clinical studies by Labanaris *et al*. [33] have shown that lymphangiogenesis increases in chronic wounds such as venous ulcers, bedsores and diabetic ulcers after treatment with negative pressure wound therapy, and an increase in LV density on histology is associated with better clinical outcomes in terms of healing rates and hospitalization time. Lymphovenous anastomosis, a microsurgical procedure for lymphedema that connects functioning LVs and veins [145], promotes DC migration and regulates chronic inflammation, which may help repair lymphedema-related lesions [146]. Cigna *et al*. [147] reported a case of lymphovenous anastomosis in a patient with refractory venous ulcers. The patient’s lymphatic leakage resolved immediately after surgery, the wound healed completely within 2 weeks and there was no recurrence after 1.5 years of follow-up. Although more clinical evidence is needed, this finding suggests the importance of restoring lymphatic drainage for chronic wound healing.

## Conclusions

Lymphangiogenesis is increasingly linked to tissue repair and regeneration, which improves tissue homeostasis, reduces inflammation and regulates immunity. Unlike angiogenesis, lymphangiogenesis during wound healing is poorly understood due to a lack of discovery and identification of lymphatic-specific markers. The structural and functional alterations of renascent LVs in wound healing, specifically chronic wounds such as venous ulcers, diabetic ulcers, bedsores and infectious wounds, must be studied in the future. Moreover, LVs are associated with HFs and peripheral nerve regeneration; however, further clarification of their relationship with these factors during wound healing is needed. Several methods for regulating lymphangiogenesis have shown that LVs can promote wound healing, and LVs should be considered in the design of skin substitutes. Additionally, drug delivery strategies that promote lymphangiogenesis need to be optimized to avoid systemic effects. It is reasonable to believe that future research will examine the function of lymphangiogenesis in wound healing and targeting lymphangiogenesis may become a novel strategy.

## Abbreviations

Angptl: Angiopoietin-like; BVs: Blood vessels; COMP: Cartilage oligomeric matrix protein; CDE: Collagen dermal equivalent; DCs: Dendritic cells; ECM: Extracellular matrix; FGF: Fibroblast growth factor; HGF: Hepatocyte growth factor; HFSCs: Hair follicle stem cells; HFs: Hair follicles; HPS: Hypoxia-preconditioned serum; IF: Interstitial fluid; IL: Interleukin; INF: Interferon; LVs: Lymphatic vessels; LECs: Lymphatic endothelial cells; LYVE-1: Lymphatic vessel endothelial hyaluronan receptor-1; lncRNA-GAS5: Long noncoding RNA GAS5; MSCs: Mesenchymal stem cells; MMPs: Matrix metalloproteinases; mBMMSCs: Mouse bone marrow mesenchymal stem cells; npBMMSCs: Neonatal porcine bone marrow mesenchymal stem cells; NMRI: Naval Medical Research Institute; PDPN: Podoplanin; PPMs: PDPN^+^ monocytes; RhoB-GTP: ras homolog family member B-guanosine triphosphate; sVEGFR-3: Soluble vascular endothelial growth factor receptor 3; TGF-β: Transforming growth factor-beta; TNF: Tumour necrosis factor; Th: T helper; Tregs: Regulatory T cells; Tie 2: Tyrosine kinase receptors with immunoglobulin and epidermal growth factor homology domains receptors 2; VECs: Vascular endothelial cells; VEGF: Vascular endothelial growth factor; VEGFR: Vascular endothelial growth factor receptor; VEZF1: Vascular endothelial zinc finger 1.
